# Hospital and Institutional News

**Published:** 1913-05-10

**Authors:** 


### HOSPITAL AND INSTITUTIONAL NEWS.

### BRITISH HOSPITALS ASSOCIATION: THE OXFORD CONFERENCE.

We take this opportunity of reminding our readers that the Fourth Annual Conference of the British Hospitals Association is to be held at Oxford from June 26 to 28. The subjects chosen for the papers include the upkeep of hospitals, the treatment of tuberculosis, and the effects of the Insurance Act. The full programme will be announced later. We hope that there will be a full attendance of members, partly because Oxford is a delightful city for a Conference and also because those who were fortunate enough to attend the previous Conference know well that, apart from the papers and formal discussions, these occasions give an extremely valuable opportunity for the exchange of experience and different views. Applications for membership of the Association should be made to the Hon. Treasurer, Mr. Stewart Johnson, Secretary of the Hospital for Sick Children, Great Ormond Street, London, accompanied by the annual subscription of half-a-guinea. Communications on the general business of the Association should be addressed to the Hon. Secretaries, Mr. Conrad Thies, and Mr. Alexander Hayes, 14 Victoria Street, Westminster, S.W.

### THE MEDICAL BOYCOTT AT SWANSEA.

Matters do not seem to improve in the unfortunate situation which has arisen at Swansea. The medical men in the neighbourhood have been roused to hostility respecting the importation of doctors from outside by the proposed local medical aid society. The honorary medical staff of the Swansea General Hospital recently resolved to boycott the " medical aid " doctors, and to refuse to treat, in the hospital, persons belonging to medical aid societies. This resolution immediately led to an outcry that the hospital should be kept out of the dispute—an end more desirable than easy of accomplishment under the circumstances. It would be too much to expect that local practitioners should be content to see other medical men imported into the neighbourhood without protest, but at the same time a hospital's staff are bound by rules which govern the admission and treatment of patients. This is clearly a case where the honorary staff and the general committee should confer, for the issues raised affect very seriously not only the welfare of the public of Swansea, but the adequate treatment and protection of the patients who may require hospital care. A hospital in the ordinary course aims necessarily at providing for the treatment of eligible patients of all types, subject to the representatives of those patients not indulging in threats or resorting to conduct which may be directly opposed to the greatest good of the greatest number. What are Dr. Le Cronier Lancaster's views.

### NEW WARD OPENED AT SWANSEA.

Last Saturday Lady Mond opened the new casualty ward at Swansea General Hospital, which has been designed by Mr. Glendenning Moxam and built by Messrs. Billings and Son. Mr. Allan Thomas, the chairman of the building committee, emphasised the need of a well-equipped casualty department for an industrial centre like Swansea, and announced that Sir Alfred Mond had given £1,000 towards its cost. It is to be hoped that the opening of this department will do something to make both parties to the dispute realise that the hospital must not be sacrificed to differences of opinion which do not affect the purpose for which it exists, and for which it has now been extended. The advice of the Mayor, Mr. David Williams, that a conference should be held to discuss the above differences, is one which we hope may be adopted, and prove the first step towards a settlement.

### INSURANCE AND DERBY ROYAL INFIRMARY.

An interesting light on the working of the Insurance Act was thrown by the report for the half-year, which Mr. Edmund Forster presented to the governors, on the progress of the Derbyshire Royal Infirmary. There has been a large increase in the number of in-patients, while those attending the out-patient department are almost as numerous as ever. The modification in the regulations relating to the admission of out-patients, which the Act's provisions has necessitated, appears to have worked well, and the following quotation is a good example of its careful and judicious phrasing: "Insured persons, being now provided with doctors, are not eligible for ordinary medical and surgical treatment in the out-patient department. They, however, will be received for consultations or special treatment when desired by their medical attendant, from whom a note to this effect must be obtained in addition to the usual out-door recommendation from a subscriber." Another point to notice is the large number of surgical cases waiting for admission, and the consequent re-discussion of the need for a new operation theatre. It is also worth while to place on record the board's answer to the inquiry of certain approved societies as to whether the infirmary would make any claim on the sick benefit of any insured person treated at the institution. In unanimously deciding to make no claim on sick benefit, the board expressed the hope that the money would be used for the benefit of the sick person concerned. Thanks, doubtless, to the tact shown by the hospital's officers in adapting their procedure to the position created by the Insurance Act, the support of the working men is still forthcoming.

### THE PUNISHMENT OF CONVALESCENT BOYS.

The Camberwell Board of Guardians have decided to support Dr. Keats, the medical superintendent at the infirmary, who had chastised a number of unruly convalescent boys. He used five strands of moderately thick string across the buttocks, the identical means he used to correct his own child. The Local Government Board, who had been notified of the facts by a visitor to the infirmary, desired the explanation of the Board. A lengthy and heated discussion resulted in the

Guardians adopting a resolution, proposed by Dr. R. Capes, that the Board expressed approval of the means taken by Dr. Keats to maintain discipline. It was stated by several members, including Mr. H. H. Edmonds, an ex-chairman, that whilst at the present juncture they had no other option but to support Dr. Keats, they were not satisfied with the mode of punishment. The Guardians, however, declined to accept a resolution, submitted by Mr. A. H. Roe, that the infirmary committee be instructed to find means to restrain refractory children without resorting to corporal punishment. While sympathising with the medical superintendent's desire for discipline, we are bound to add that the course he has adopted, and that has received the approval of the Guardians, sets a precedent which is very easily liable to grave abuse. Once corporal punishment is permitted in a hospital for one officer, junior officials, who may always include a quick-tempered nurse, are apt to give up that self-control which is imperative on all where corporal punishment is absolutely forbidden.

### THE ROYAL MEDICAL BENEVOLENT FUND.

At the festival dinner in aid of the Royal Medical Benevolent Fund, which, it will be remembered, received only last year his Majesty's permission to the style "Royal," it was announced that the King had consented to become the Fund's patron. Sir James Tweedy, who presided in the absence of Prince Arthur of Connaught, read a statement from the latter reminding the public that though fame and fortune are the prizes won by a few of the profession, the rank and file add to a long and arduous training the risk of going to the wall through ill-health or other causes. "Even those whose talent and industry give promise of a brilliant career," the Prince remarked, "may be struck down by illness, and it is to provide for the fatherless children of such" that the Fund counts among its cherished objects. He referred also to the gratitude of the Royal Family and himself to the profession, for their skill and care in attending the Duchess of Connaught, and he and the Duke each sent a cheque for £25 to the Fund. Someone has said that there are few more tragic things than a sick doctor, and there is certainly no profession which excites more sympathy when illness and its disastrous consequences attends the doctor and his family.

### HOSPITAL AND SCHOOL COMBINED.

The Sheffield Education Committee have agreed to two important proposals. In the first place, the open-air school at Whiteley Woods is to become a boarding school—that is to say, the tuberculous children who attend it will not return home at night, but will sleep at the institution. When the open-air school at Paddington was opened some time ago the authorities then let it be known how greatly the benefit which the children received during the daytime was mitigated by the unsuitable conditions which they experienced in their homes at night. The residential principle, then, is the right one from the children's point of view, but the ratepayers are apt to object to the necessary expense. The second proposal agreed to is the taking over of the King Edward VII. Memorial Institution for Crippled Children, which will be under the care of the Education Committee, and will be largely for those suffering from tuberculous joints, whom Dr. Ralph Williams has stated can be cured if taken in time. "A large proportion of these children will necessarily be in bed," he states in his report as medical officer. "Teachers, however, will be provided by the Education Committee." The above are two good examples of the hospital and school combined—the latest extension of the former.

### AN ADEQUATE RAPID AMBULANCE SERVICE.

Great emphasis is laid by the City Coroner upon the need for an adequate rapid Ambulance Service similar to that which has worked so well in the City for the past five years or so. The importance of this was indicated by the evidence at the inquest upon the body of a workman found on the footway of Tower Bridge, reference to which was made in The Hospital the other day. It is unfortunate that, as stated in Dr. Waldo's report, owing to a difference of opinion with regard to the recommendation of the Departmental Committee, the County Council obtained an Act of Parliament in 1909 giving them power to establish a rapid ambulance for London outside the City, and whilst, so far, nothing practical has resulted from the Act, it has had the effect of prejudicing a much-needed reform. The Departmental Committee recommended that the Metropolitan Asylums Board should be authorised by Act of Parliament to apply their funds to the establishment of a service of non-infectious ambulances for the transport of street cases and generally to co-operate with the Metropolitan Police. The Asylums Board has had considerable experience in ambulance work, and such an arrangement would relieve the somewhat overburdened Council from the discharge of so pressing and important a duty.

### MR. WELLCOME'S MEDICAL MUSEUM.

The day of medical museums appears to be approaching. A historical medical museum, organised by Mr. H. S. Wellcome, is announced to be opened at the end of June. It will aim at a popular as well as a scientific list of visitors, for, in addition to ancient miscroscopes and amulets connected with English folk medicine, there will be a section to include some of the instruments used by famous surgeons when operating on historical personages. We shall hope to examine the collection, and until it is on view it would be unfair to estimate its interest or value. The original apparatus used by Galvani in the eighteenth century will be instructive to those interested in the devolopment of scientific instruments.

### THE DEATH OF MRS. D'OYLY CARTE.

We have often had occasion to draw attention to the good fellowship and personal service which the profession of acting, and, indeed, all branches of the theatre, display in the service of the hospitals and the sick. Mrs. D'Oyly Carte, or Mrs. Stanley

Boulter as she became in 1902, was always a friend to the hospitals, and only last year the King conferred on her the Order of Mercy. Her death this week, after a long illness, removes a figure well known to a large public and to many famous persons, Whistler among them; and the Hospital for Sick Children, Great Ormond Street, has good cause to remember her. Thirteen years ago the leading theatrical managers in London desired to present her with her portrait, but she asked that the money collected might be used instead to found a bed in the Children's Hospital for the benefit of the Actors' Benevolent Society.

### THE FATHER OF THE COLLEGE OF SURGEONS.

Two months ago it was noticed by the press that the senior member of the Royal College of Surgeons, Surgeon-Major Henry Benjamin Hinton, had attained his hundredth birthday; and the *Australian Medical Gazette* supplies some very interesting further details about this gallant centenarian. Major Hinton joined the Bengal Army soon after he qualified, and saw active service in the Indian Mutiny, in China, and in Burmah. In 1868 he retired on a pension, and settled in Australia, where he has since resided. At the age of eighty-six Major Hinton went to South Africa, being anxious to offer himself for the medical service in the Boer War; but the age limit frustrated his plucky intention. His faculties are excellent, and his memory is as keen as his interest in every contemporary movement. The progress of Australia is, we are given to understand, especially dear to his heart. About sixteen months ago he unfortunately fractured one thigh bone, which accident has considerably curtailed his bodily activities. By a very remarkable coincidence Major Hinton's immediate predecessor in the "fatherhood" of the College of Surgeons was another Hinton, also an ex-major of the Indian Service; but no relationship existed between the two. This latter veteran survived to the age of 104.

### THE SALARIES OF HOSPITAL ALMONERS.

At the recent quarterly meeting of the Radcliffe Infirmary and County Hospital, Oxford, it was decided to raise the salary of the lady almoner, Miss Linton, from £50 to £75 per annum. More than one speaker paid a tribute to the value of the work and to the tact with which Miss Linton had carried it out. It is interesting to note in this connection that, in spite of the Insurance Act, the out-patients have only decreased by 47, while the casualties have increased by 222. That being so, and the principle of the almoner admitted, especially in its constructive aspect of "following up" and after-care, the skilled work required and its success seemed to demand a scale of pay reflecting these qualities.

### HOSPITAL MEMORIAL TO ETHERINGTON-SMITH.

At a meeting held at St. Bartholomew's Hospital on May 1 it was decided to open a subscription list for a memorial to the late Mr. R. B. Etherington-Smith. It is suggested that the memorial should take the form of providing and endowing separate sick quarters at St. Bartholomew's Hospital for the use of the medical, surgical, and resident staff, and of providing a suitable memorial at Cambridge. "Etherington-Smith's life work," write the representatives of St. Bartholomew's Hospital and the University Boat Clubs who are supporting the proposal, "was so bound up with St. Bartholomew's that this form of memorial will doubtless commend itself not only to his colleagues in the profession, but also to the larger body of men who knew and admired him as an oarsman and friend. The scheme has received the cordial approval of his family." Subscriptions will be received by the Hon. Treasurer, Dr. T. W. Shore, Dean of the Medical School, St. Barthlomew's Hospital, E.C.; or they may be sent to "The Etherington-Smith Memorial Fund," at the Bank of England.

### FRIENDLY SOCIETIES AND PANEL DOCTORS.

Notwithstanding an authoritative statement from the Insurance Commissioners to the effect that sickness benefit would issue on a medical certificate signed by any doctor, whether on the panel or not, a branch of the Ancient Order of Foresters thought fit to pass a resolution that only certificates from panel doctors should be accepted. This abominable rule was put into force to the prejudice of Mr. W. V. Heard, a member of the society, who was refused his benefit because the certificate supporting his claim to it was signed by a non-paneller. With commendable public spirit Mr. Heard brought an action against the trustees of this approved society, which Mr. Justice Bailhache decided against him on technical grounds. However, the case has been carried to the Court of Appeal, where three Lords Justices have unanimously set aside the decision of the lower Court, and have declared the Foresters' resolution to be *ultra vires*, illegal, and unenforceable. We congratulate Mr. Heard on this decision, which puts an end to a great injustice. To be compelled by law to pay insurance premiums and then to be refused sick pay because he preferred to employ a non-panel doctor was indeed a piece of tyranny worth resisting.

### GUY'S HOSPITAL NEW BUILDINGS.

Mr. A. J. Balfour, who is a governor of the hospital, has consented to open the new buildings at Guy's Hospital on June 3. They consist of a new pathological department, a new school of dentistry, and new chemistry and physics laboratories. The proceedings will include a luncheon, and the opening ceremony will be followed by a garden party, when the new school buildings will be open to the inspection of visitors.

### THE GUY'S SURGICAL "AT HOME."

On Monday last the surgeons of Guy's Hospital gave an "At Home" to the surgeons of the other London hospitals. About forty were present. About four hours were spent in the different theatres watching operations performed by the surgeons of Guy's. Mr. Lane's operations, which were well attended, comprised colectomy, appendix controlled by ilium, and malunited fractured radius and ulna. The new theatres which were opened a year ago were then inspected, and at 4 P.M. the tea was served in the Court Room. The visitors expressed themselves especially pleased with the efficient way in which the surgeons' assistants performed their part.

### THE LEVER PATHOLOGICAL MUSEUM.

On Tuesday evening the Pathological, Bacteriological, and Serological Museum, which Sir William Lever's generosity has presented to the Royal Institute of Public Health, Russell Square, was inaugurated by Lord Beauchamp. The aim of the Museum is a complete collection of the micro-organisms connected with the more important infectious diseases. Professor W. D. Smith introduced Sir William Lever, who handed over the Museum to Lord Beauchamp, the president of the Institute. Demonstrations in the different departments of the Institute's work were given during the evening.

### TRIBUTE TO A HOSPITAL TREASURER.

Mr. H. Brown, honorary treasurer of the Watford District Hospital, received a very warm tribute from the Chairman of the Committee, Mr. E. H. Loyd, at the annual meeting. A deficit of £160 had been converted into a balance in hand of £14, while several new annual subscribers had been added, and the reason of this was due, said Mr. Loyd, to the immense amount of time and trouble which Mr. Brown had taken. The Hospital Saturday Fund has fallen by nearly £100, and no doubt the new secretary, Mr. A. S. Fisher, has found it difficult to retain the hold of the institution on this Fund since the Insurance Act has come into force. The Chairman made a first-rate speech, showing how greatly the hospital's work had increased, and how the strain on the staff had led to new appointments—two additional honorary medical officers and a new staff nurse. We emphasise this because the attendance was very small, which once again raises the question of what practical use, apart from its formal necessity, can the annual meeting be made to be in increasing the support and enthusiasm in the neighbourhood.

### DEATH OF A HOSPITAL FOUNDER.

A special meeting of the committee of management of Belfast Nervous Diseases Hospital has been held to record the loss to the institution by the death of its founder, Dr. John M. MacCormac. Sixteen years ago he founded the institution, and it has been his chief interest and care, inspiring him to much active work in the capacity of its honorary physician. Mr. Frank Workman, the hospital's president, recounted Dr. MacCormac's services, and the Rev. Dr. Purves paid a tribute to his memory, which was based upon personal as well as public grounds. Dr. MacCormac, who qualified in 1867, was a graduate in medicine of Durham University. One of the last acts of his life was to draw up the report of the cases treated by him for the purpose of presentation to the approaching annual meeting.

### MEDICAL MISSIONARIES.

*Apropos* of our recent comments upon Medical Missions, a correspondent points out to us that the monument to Livingstone in Westminster Abbey contains absolutely no reference in a somewhat lengthy inscription to the fact that the explorer was a qualified medical man. It appears also from a passage of autobiography in one of Livingstone's books, quoted by the *Glasgow Medical Journal*, that he was very nearly rejected by the examiners of the Faculty of Physicians and Surgeons of Glasgow at his final examination on account of his championship of the stethoscope as a help to diagnosis! It is to be presumed that in London, where the explorer had been studying, the cult of this French invention had progressed a good deal further than in Glasgow, and that Livingstone's obstinacy in sticking to his opinions put the examiners in a bad humour. While on the subject of medical missionaries, we may note that Dr. J. A. C. Smith has discovered seven new species of mammals on an expedition into the far interior of China. Dr. Smith was formerly a medical missionary, but has, we believe, abandoned that sphere of work in favour of zoological science and exploration in little known districts of China.

### A REMARKABLE PROVINCIAL TREASURER.

Mr. W. Precey has been re-elected treasurer of the Walsall and District Hospital, a position which he has held for twenty-three years. This long tenure of office, however, does not by any means exhaust Mr. Precey's connection with the hospital, which extends to the remarkably long period of thirty-eight years. One of the features of his treasurership was the introduction some years ago of the Uniform System of Hospital Accounts. It is one of the best features of the voluntary system that it offers to individuals, and on occasion, indeed, to members of the same family, an opportunity of personal service which extends to the best part of their lives, and Mr. Precey's thirty-eight years of service is a good example of such an opportunity acted on to the full. He receives not merely the congratulations of his hospital, but of that world of institutional workers which is always ready to note with friendly rivalry the personal records of its colleagues in hospital and institutional work.

### PUBLIC APPOINTMENTS AND THE RIGHT TO MARRY.

The Women's Freedom League has been corresponding with the London County Council in respect of the appointment of two women doctors in the Council's Health Department "under the condition of resigning should they marry." Mr. Cyril Cobb, the Chairman of the County Council, has received from the Secretary of the League a protest against "the tyranny and impertinence of this constant interference with the rights of women citizens in all spheres of public life." In answer to a request that the Council should receive a deputation on the matter, Sir Laurence Gomme replied that the question was determined on, after very full consideration, and that the deputation could serve no useful purpose. It is important to notice that the above restriction is not on a par with that which some public bodies impose on married couples not to have children, as the phrase "without encumbrance" means. And until sufficient numbers of women doctors are appointed to the above posts we fear that public bodies will not change their opinion. Married women are liable to prolonged absences from work which at present no means appears to have been found for supplementing. It is very unfortunate, but sex disability is a fact of Nature which has not yet been got over by any satisfactory plan.

### PANEL SERVITUDE IN LONDON.

We are glad to note that Mr. Hunt, M.P., has asked a question in the House concerning the fact, alluded to in our columns last week, that 106 panel practitioners in London have from two to six or seven thousand patients on their lists. In reply Mr. Masterman stated that the London Insurance Committee had informed him that no doctor on the London panel had 7,000 on his list. "Some misapprehension," he added, "has been caused by the fact that, in certain cases, the doctor who is supposed to have a large number on his list is in fact the senior member of a partnership or firm, the members of which are collectively responsible for the treatment of those persons." That is all very well as far as it goes, but that it does not go far as an explanation is proved by the fact that the Insurance Committee has so far refused to limit the number of patients which a panel doctor may place on his list. Such a limit is the only way in which a condition of things worse than club practice can be prevented. Things have been bad enough already for the unfortunate insured person, but what they will become in the case of an epidemic it is dreadful to think.

### CENTRAL BRANCH AT MANCHESTER ROYAL INFIRMARY.

A central branch of the Royal Infirmary for accidents and out-patients is now being built at Manchester, the erection of which was one of the conditions attached to the removal of the institution. Since that took place the old out-patient department has been used as a temporary accident room. The new branch will also contain twelve beds for cases of serious accident. Great interest has been aroused in the appeal for £25,000 to pay off the cost of the building, since the King and Queen will visit Manchester in July, and it is hoped that the buildings may be rid of debt at that date, as the new infirmary was on the occasion of its opening by King Edward and Queen Alexandra. The new unit will be a comprehensive one on a scale worthy of the great institution to which it will be attached. The Manchester public, which subscribed the £100,000 asked for the new infirmary in a little over twelve months, has here an opportunity to complete the institution then reconstructed.

### PARENTS AND VIVISECTION.

We cannot congratulate Dr. Arthur Westerman, however much we may commend his candour, upon making his little daughter, aged eleven months, the subject of an experiment on the effect of small doses of boric acid. His action has been rendered infinitely more distasteful and astonishing by the advertisement given to it in the *Daily Mail* and some other papers. These have published a photograph of the baby experimented upon. If Dr. Westerman considers that his conduct in this matter calls for explanation, it should surely have been made to the authorities of his University and the authorised representatives of his profession. How can he justify the publication of the details of his experiment and a defence of his procedure in the daily papers? Dr. Westerman's defence that as a qualified man he has a right to experiment on his children cannot be admitted. The greater the knowledge and power entrusted to a man in regard to issues which affect life and death, and health and illness, the greater call is there to insist that the only human being he is justified in submitting to experimental treatment is himself. We hope this is the last occasion upon which the daily papers will give publicity with illustrated details to an action of this kind. For it is the first time, we believe, in the history of the world that a member of the medical profession has claimed notoriety and credit for himself for making his baby child the subject of experiment.

### FURTHER SANATORIUM PATIENTS' COMPLAINTS.

The quality and quantity of the food at the Strinesdale Hospital, which is being used for the treatment of consumptives, has been complained of by two discharged patients. The sanatoria subcommittee of the Oldham Insurance Committee therefore appointed two of their members to inspect and report on the matter. The result of their investigation was to the effect that no complaints were made by the patients in either the men's or the women's ward; and that the metal teapots complained of in the former have been exchanged for porcelain ones. Want of privacy in the bathroom was complained of, but the visiting members found that the clear window could be covered by a curtain, which they state is always kept behind the door in the room. As regards the absence of a lock on the bathroom door, the visiting members recommend that it should be left as at present—a point of view that all acquainted with hospital administration will uphold. In conclusion, the report states: "Our opinion is that the complaints emanated from persons naturally of an irritable and complaining nature. This, we think, is a bad feature in an institution of this kind. We find, however, that the complaints were entirely without foundation, and calculated to bring the sanatorium into disrepute if this class of patient is not kept under strict discipline." There seems to have set in a fashion among patients at institutions of this kind to complain of late. The moral is, as we have often insisted, to be extremely careful to appoint a medical superintendent who has had full practical experience of the administration of consumptive patients.

### THE EPILEPTIC CHILDREN OF LEEDS.

In reporting to the Education Committee on the health of the elementary school children in Leeds, Dr. Algernon Wear draws attention to the fact that "there is no local colony or home for the large number of epileptic children in the city who are receiving no education or training." Leeds, which has shown such public spirit in maintaining the efficiency of the general infirmary, might well take a lesson from the Monyhull Colony for Epileptics and do something for the training of the epileptics whose condition at present Dr. Algernon Wear so rightly deplores. The example has been set and the way shown for dealing with this problem. We hope that Dr. Wear's hint will not fall upon deaf ears.

### MEDICAL OFFICER ASKED TO RESIGN.

The Doncaster Rural District Council have called upon their medical officer of health, Dr. A. B. Dunne, to resign. The climax to a certain want of harmony, which is now alleged to have existed in the Council for some time, arose over a letter sent to the sanitary committee by one of the sanitary inspectors. This letter referred to the administration of the public health department, and led to the appointment of a sub-committee, who presented a brief report to the Council last week, and recommended with one dissentient that Dr. Dunne be asked to resign. This recommendation was adopted by the Council by twenty-one votes to two. Dr. Dunne, who is an M.B., B.Ch., of Cambridge, and holds the Conjoint Diploma, came to Doncaster nearly two years ago, and was previously medical officer to the Nottingham Education Authority. It is also "recalled" locally that Dr. Dunne's predecessor, as medical officer, was unable always to work smoothly with the Council.

### AUSTRALIAN MEDICAL BUREAU IN LONDON.

The Federal Government has created a Commonwealth Medical Bureau in connection with the office of the High Commissioner in London. The primary purpose of the Bureau is to ensure the thorough medical examination of all intending emigrants to Australia before they are allowed to leave any British port. A chief Medical Officer being required to take charge of this new institution, Dr. W. P. Norris, having lately resigned his post as Director of Federal Quarantine, applied for and obtained the London position at a sacrifice of £200 per annum salary. His task will be to organise the Bureau and set up a system of medical inspection at all ports from which emigrants leave the United Kingdom for the Commonwealth. Dr. Norris will supervise the work of the medical examiners and keep in touch with the Board of Trade officers who carry out the provisions of the Merchant Service Act. Dr. Norris is forty-seven, and holds the degrees of M.D. and Ch.B. (Melb.), the Diploma of Public Health and other qualifications (Eng.). He was in private practice for some years, and in 1900 became assistant medical inspector under the late Dr. D. Astley Grosswell, whom, later, he succeeded as chief of the State Health Department. In 1909 he was appointed to the position he has just left.

### INSTITUTIONAL POULTRY FARMING.

The suggestion made by one of our correspondents that unoccupied land adjoining the premises of hospitals and similar institutions should be utilised for the purpose of poultry farming is worth more consideration from institutional housekeepers than it receives. To those—and there are many—who have experienced the difficulty of obtaining a reliable supply of fresh eggs at a reasonable or indeed at any price, the practical application of this suggestion ought to be acceptable. The commissariat of many institutions have had sufficient foresight to appreciate the importance of this fact, but the practice of institutional poultry farming is by no means general. In a few institutions we are acquainted with, the gardener is allowed the privilege of keeping chickens in the grounds adjoining his lodge, upon the condition that he supplies eggs and table birds to the institution at reasonable prices. Whilst an arrangement such as this relieves the matron or officer, responsible for the housekeeping, of a certain amount of trouble, it points to a lack of enterprise on their part. From our knowledge of institutional managers we are sure that, generally speaking, they are only too glad to welcome suggestions, and to give a great deal of personal assistance as well, in putting into practice useful ideas. There must be more than one, at least, amongst the female staffs of every institution who would be glad to have the relaxation which the keeping of poultry would afford, and a sufficient knowledge of the subject could quite easily be acquired. As a use for unoccupied land it deserves attention.

### THIS WEEK'S DRUG MARKET.

Few changes in value have occurred during the week, and business in the Drug market has been far from active. Very little business has been done in cod-liver oil, and the value still has a downward tendency. Ergot of rye is, if anything, a trifle cheaper. Business has been done in essence of lemon at firm prices. Opium, morphine, and codeine are unchanged in position, but values are maintained. Oil of orange is rather dearer, as also is citric acid. Cape aloes is scarce and tends dearer. Cardamoms are slightly lower in price, as also are calumba root and sarsaparilla.

### OUR HOSPITAL SUNDAY SPECIAL NUMBER.

We hope to publish on the 14th inst., as usual, in the week before before Hospital Sunday, which falls this year on May 25, a Special Number of The Hospital, dealing with the whole question, which will be distributed to the congregations of London on May 18. The needs of the London Hospitals and the position of the Fund will be discussed in several important articles.

